# Comparison of tamoxifen and hormone replacement cycle (HRT) in frozen embryo transfer. A randomized controlled trial

**DOI:** 10.5935/1518-0557.20220078

**Published:** 2023

**Authors:** Rahele Ebrahimi, Malihe Mahmoudinia, Nayree Khadem, Sedighe Rastaghi, Behnaz Souizi, Tahereh Sadeghi

**Affiliations:** 1 Maternal & Neonatal Research Center, Faculty of Medicines, Mashhad University of Medical Sciences, Mashhad, Iran; 2 Department of Biostatistics, School of Health, Mashhad University of Medical Sciences, Mashhad, Iran; 3 Department of Obstetrics & Gynecology, School of Medicine, Sabzevar University of Medical Sciences, Sabzevar, Iran; 4 Assistant Professor of Nursing, Nursing and Midwifery Care Research Center, Mashhad University of Medical Sciences, Mashhad, Iran

**Keywords:** tamoxifen, IVF, GnRH agonist, frozen embryos transfer, endometrial thickness

## Abstract

**Objective:**

The use of frozen embryos in the treatment of infertility with assisted reproductive techniques has been increased. Different methods are used to prepare the endometrium for frozen embryo transfer (FET). The aim of this study was to compare pregnancy outcomes after treatment with tamoxifen and hormonal replacement therapy (HRT) in FET.

**Methods:**

This randomized clinical trial was carried out with 214 infertile women in the infertility research center of Milad Hospital in Mashhad during 2018-2020. We had 84 patients receiving tamoxifen and 92 took HRT. Endometrial thickness (ET) and pregnancy outcome were measured in both groups.

**Results:**

Mean infertility duration (*p*=0.328), number of embryos (*p*=0.649), FSH (*p*=0.390), LH (*p*=0.051) and LH/FSH ratio (*p*=0.287) as well as type of infertility (primary or secondary) (*p*=0.295), causes of infertility (*p*=0.750) and pattern of menstruation (*p*=0.676) were not significantly different in the two groups. Mean ET in the TMX and HRT groups were 8.72±1.45mm and 9.00±1.69mm, respectively (*p*=0.423). There was no statistically significant difference between chemical pregnancy (*p=*0.663), clinical pregnancy (*p*=0.994) and ongoing pregnancy (*p*≥0.999) in the TMX and HRT groups.

**Conclusions:**

Treatment with tamoxifen can be as effective as GnRH agonist for endometrial preparation in FET.

## INTRODUCTION

Frozen embryo transfer has become a common method in the *in vitro* fertilization (IVF) cycle (Khadem Ghaebi *et al*., 2020). Its use has increased by the improvement of cryopreservation techniques over the past years. Pregnancy and live birth are comparable with fresh cycles or even better than it (Zhu *et al*., 2011; Roque *et al*., 2013; Wei *et al*., 2019; Chen *et al*., 2016).

Proper synchronization between the fetus and endometrial growth is an important factor in success in IVF (Chen *et al*., 2016; Nikzad *et al*., 2013; Wirleitner *et al*., 2016; Gomaa *et al*., 2015). Successful implantation depends on the adequate growth of the endometrium; therefore consideration of the thickness and pattern of the endometrium is an essential factor for FET (Gingold *et al*., 2015; Eftekhar *et al.*, 2018). Thin endometrium is associated with poor pregnancy outcomes in assisted reproductive technology (Chen *et al*., 2010).

Different cycle protocols are used to prepare the endometrium for FET, including a natural cycle, a hormone replacement cycle (HRT), or a stimulated cycle (Khadem Ghaebi *et al*., 2020; Eftekhar *et al*., 2018; Ghobara *et al*., 2017; Mackens *et al*., 2017). Natural cycles are commonly used in women with regular menstrual cycles. In the HRT protocol, the endometrium is prepared by estrogen and progesterone with or without the GnRH agonist. Stimulation with gonadotropin or letrozole is usually suggested for women who have irregular menstrual cycles or patients who did not respond to hormone replacement treatment in a previous cycle (Azimi Nekoo *et al*., 2015; Cerrillo *et al*., 2017; Aleyasin *et al*., 2017; Yarali *et al*., 2016). Each of these protocols has its benefits and disadvantages. So far there is no standard method and no method is preferable to another.

Tamoxifen is a selective estrogen receptor modulator (SERM) that it’s approved as a highly effective agent for the prevention and treatment of breast cancer (Pourmatroud *et al*., 2013). In addition, tamoxifen has been used to induce ovulation (Reynolds *et al*., 2010; Seyedoshohadaei *et al*., 2012). Tamoxifen has an agonist effect on estrogen receptors in the endometrium during induction of ovulation and, as a result, it increases endometrium thickness. Therefore, it can increase live births in the endometrial preparation cycle in FET. Previous studies in patients with thin endometrium have revealed that successful implantation rates improved after treatment with tamoxifen in FET (Chen & Chen, 2013; Tian *et al.*, 2015; Ke *et al*., 2018) Therefore, the aim of this study was to compare endometrial thickness and clinical pregnancy outcomes in hormonal replacement therapy or tamoxifen and gonadotropin for endometrial preparation in FET.

## MATERIALS AND METHODS

### Study design

This study was a double-blind randomized clinical trial, performed on 214 infertile women in IVF with frozen embryos, referred to Milad Infertility Center, affiliated with Mashhad University of Medical Sciences, Mashhad ,Iran , from the beginning of June 2019 to the end of September 2020. This study was approved by the Medical Ethics Committee of Mashhad University of Medical Sciences on 28 January 2019 with letter number IR.MUMS.MEDICAL.REC.1399.003 of 98. It has also been registered in the Clinical Trial Registration Center of Iran under the IRCT20181030041503N3 number.

### Participants

Infertile women (18-42 years old) who were candidates for IVF with frozen embryos with no history of severe male infertility, severe endometriosis grades 3 and 4, primary and secondary amenorrhea were included in the study, those patients willing to participate. Patients with necrotic and low-quality embryos after thawing or having no live embryo after the thawing procedure were excluded from the study.

The sample size was calculated on basis of a pilot study. The frequency of chemical pregnancies calculated was 0.35% in the control group and 0.55% in the intervention group. In this study, the minimum sample size, keeping an alpha error of 0.05 and a beta equal to 0.2, was calculated to be 196 people (98 subjects in each group). Due to the probability of sample dropout rate, about 10% were added to the above number and the final sample size was 107 people in each group.

The 214 women were divided randomly into two groups. Random allocation was performed using a computer generated random list. The researcher who did sonography as well as the analyst did not know the treatment of patients and groups. It was not possible for patients to be blind, because the type of drugs prescribed to them (injectable or oral) were different in the two groups.

### Endometrial preparation

Patients were allocated randomly into two groups. The first group, cycle stimulated with tamoxifen plus human menopausal gonadotropin (HMG) (TMX group); and the second group implemented routine artificial hormonal endometrial preparation (HRT group).

The first group received tamoxifen 20 mg (Iran Hormone Pharmaceutical Co, Tehran, Iran) twice-a-day from the third day of the cycle for five consecutive days, and HMG (Menotropin, Darou Pakhsh Pharmaceutical Co, Tehran, Iran) was prescribed intramuscularly at a dose of 150 mg on days 6 and 8. The next evaluation of patients was performed on days 10 of the cycle with transvaginal ultrasound to measure follicle diameter and endometrial thickness. The stimulation with HMG was given until the follicular size reached 18mm. Vaginal ultrasound scans were performed every 3 days until the endometrial thickness was ≥8 mm and the dominant follicle diameter was 18mm; then, HCG 10000 IU (Choriomon, IBSA, Lugano, Switzerland) was injected to induce ovulation. If the endometrial thickness did not reach 8 mm after 15 days, the cycle would be cancelled regardless of the follicle diameter. Vaginal progesterone 200 mg (Utrogestan, Besins, Brussels, Belgium) was used three times a day, 48 hours after the HCG injection. FET was performed on the fourth day of progesterone use. Luteal support with progesterone continued if the βhCG test was positive at least for 8 weeks, otherwise it was discontinued immediately after a negative pregnancy test.

In the HRT group, oral contraceptive-LD (manufactured by Aburaihan Co., Tehran- Iran) was taken on the fifth day of the previous cycle and continued for 21 days One-third of decapeptil depo 3.75 (Decapeptyl 3.75, Ferring, Germany) was administered on the 21^st^ day of the cycle (Midluteal). At the begging of menstrual cycle, trans-vaginal ultrasound (TVU) was done to confirm pituitary desensitization if the ovaries were quiet and endometrial thickness was less than 5 mm, oral estradiol valerat (manufactured by Aburaihan Co., Tehran-Iran) was prescribed at a dose of 2 mg twice-a-day and was increased to 2mg three times a day after three days. On the 12^th^ day, TVU was performed to monitor endometrial thickness. The dose of estradiol was adjusted according to endometrial thickness to reach a maximum of 8 mg per day. Vaginal sonography examinations were repeated every 3 days. When the endometrial thickness reached 8 mm with a triple line pattern, vaginal progesterone 200mg (Utrogestan, Besins, Brussels, Belgium) was started three times a day. Embryo transfer was performed on the sixth day of progesterone use. Estrogen continued at the same dose until 8 weeks of pregnancy. All ultrasound examinations were performed by an expert researcher using a Phillips ultrasound affinity 70 device at the Milad infertility center.

In both groups, luteal support continued until the result from the pregnancy test. If pregnancy occurred, the drugs continued till 8 weeks of gestation.

### Embryo thawing

The cleavage-stage embryo was placed on the tip of the Cryotop (Kitazato Corporation, Tokyo, Japan). All cryotop were separated from the liquid nitrogen 48 hours before the planed transfer and were quickly immersed into the thawing solution and left there for 1 minute at room temperature then the embryo was aspirated using a pipette and gently placed on the bottom of the dilution solution for 3 minutes and at the end, it was placed in washing solution for 5 minutes in 2 step. The 5-day-old embryo was cultured in Blastocyst-media (Origio, Denmark). Blastocyst embryos were assessed according to Gardner’s criteria based on inner cell mass, blastocyst expansion, and trophectoderm development. One or two embryos of top quality under Phillips transabdominal ultrasound (affinity 70) were transferred by Cook catheter (Cook, Medical, and Eight Mile Plains, Queensland, Australia). When there was no blastocyst on day 5, the most advanced embryo was transferred.

### Outcome measurements

Our primary outcomes were chemical pregnancy, clinical pregnancy. BHCG serum was measured 14 days after embryo transfer. Clinical pregnancy rates defined as the observation of at least one fetus and heartbeat on transvaginal ultrasound after the 42-day transfer.

The secondary outcome was ongoing pregnancy, determined by a 12-week or longer pregnancy; and also endometrial thickness was measured on the day of progesterone administration. The endometrial measurements were done by two researchers with similar criteria. They measured it in the thickest part of the uterus on sagittal views, and it was repeated at least 3 or 4 times.

### Statistical analysis

In order to examine the quantitative data in terms of having a normal distribution, we used the Shapiro-Wilk test. The mean standard deviation was used to describe the individual-personal information and personal characteristics. We employed the Mann-Whitney test to compare quantitative variables between the two groups due to abnormal data distribution. Qualitative data were described in terms of frequency and percentage; and they were analyzed by the Chi-square or Fisher’s exact test. The Statistical Package for Social Sciences (SPSS®) version 20 was used for data analysis. A *p*-value <0.05 was considered statistically significant.

## RESULTS

**Figure 1:**
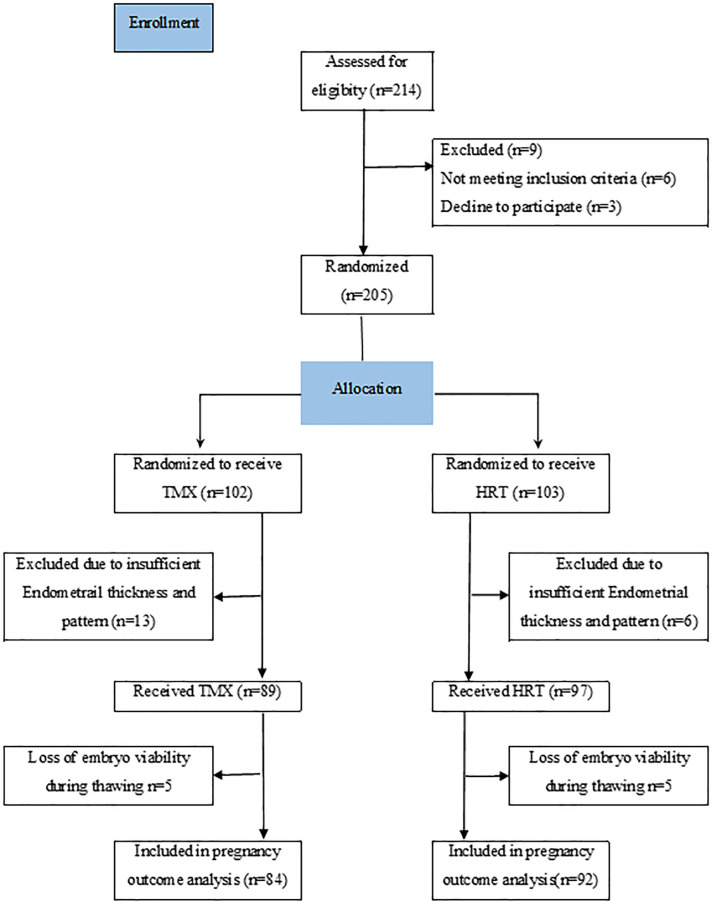
CONSORT flow diagram of the study.

214 patients, 107 in the TMX group and 107 in the HRT group (GnRH agonist and estradiol valerate) were included initially in the study. Nine patients were excluded from the study due to not meeting inclusion criteria and lost to follow-up (5 in the TMX and 4 in the HRT group). In addition, 19 participants (13 in the TMX and 6 in the HRT groups) were taken off treatment due to insufficient endometrial thickness and pattern; and finally, 11 patients because of lack of embryo viability during thawing were excluded (5 from each group). Eventually, 176 participants completed the treatment and entered our analysis (Figure 1).

The mean age of patients in the TMX group was 31.07±5.66 years and in the HRT group was 29.95±4.99 years (*p*=0.172). The mean BMI of patients in the tamoxifen group was 24.77±3.92kg/m^2^ and in the HRT group it was 24.96±3.54kg/m^2^ (*p*=0.903). Additionally, the mean duration of infertility in the TMX group was 5.52±3.67 years and in the HRT group it was 5.89±3.52 years (*p*=0.328). Also, the mean FSH (*p*=0.390), the mean LH (0.051) and LH/FSH ratio (*p*=.287) of patients in the study groups were not statistically significant. In addition, the Chi-square test showed no statistically significant difference between the two groups in terms of the types of infertility (primary and secondary) (*p*=0.295), cause of infertility (*p*=.750), regular and irregular menstruation (*p*=0.750) (Table 1). Furthermore, the Chi-square test showed no statistically significant difference between the rate of chemical pregnancy (*p*=0.663), clinical pregnancy (*p*=0.994) and ongoing pregnancy (*p*=0.423) in the HRT and TMX groups. There was no statistically significant difference in the rate of cycle cancellation in both groups (*p*=.098), so the rate of cycle cancellation in the TMX group were significantly higher than in the HRT group. The mean endometrial thickness of patients in the TMX group was 8.72±1.45 mm and in the HRT group it was 9.00±1.69mm, which was not statistically significant (*p*=0423) (Table 2).

**Table 1:** Comparison of demographic and hormonal characteristics of study groups.

Variables		TMX n=84	HRT n=92	*p*-value
Age (years)		31.07±5.66	29.95±4.99	0.172^b^
BMI (kg/m^2^)		24.77±3.92	24.96±3.54	0.903^b^
Duration of infertility (years)		5.52±3.67	5.89±3.52	0.328^b^
Basal level FSH (mIU/ml)		6.09±2.08	5.89±2.30	0.39^b^
Basal level LH (mIU/ml)		7.07±4.57	6.47±4.51	0.051^b^
LH/FSH (mIU/ml)		1.31±1.04	1.24±0.98	0.287^b^
Number of embryo		2.19±0.66	2.26±0.79	0.649^b^
Type of infertility	Primary Secondary	61 (72.6%) 23 (27.4%)	74 (80.4%) 18 (19.6%)	0.295^a^
Causes of infertility	Unexplained Ovulatory Tubal Uterine Male factor Mix factor More than one female factor	18 (21.4%) 22 (26.2%) 10 (11.9%) 1 (1.2%) 18 (21.4%) 13 (15.5%) 2 (2.4%)	22 (23.9%) 22 (23.9%) 5 (5.4%) 3 (3.3%) 22 (23.9%) 15 (16.3%) 3 (3.3%)	0.750^a^
Pattern of menstrual cycle	Regular Irregular	55 (65.5%) 29 (34.5%)	64 (69.6%) 28 (30.4%)	0.676^a^

a Chi-square test

b Mann-Whitney test

TMX: Tamoxifen, HRT: Hormone replacement therapy, BMI: body mass index Weight⁄(Height)^2^, FSH: follicle stimulating hormone, LH: luteinizing hormone.

**Table 2:** Comparison of cycles and pregnancy outcomes between groups.

	TMX n=84	HRT n=92	
Canceled cycles Total - Insufficient Endometrial thickness - Pattern endometrial - Liquid in Endometrial	13/84 (15.5%) 5 5 5	6/92 (6.5%) 6 0 0	0.095
Chemical pregnancy	23 (27.4%)	29 (31.5%)	0.663^a^
Clinical pregnancy	22 (26.2%)	23 (25.0%)	0.994^a^
Ongoing pregnancy	13 (15.5%)	15 (16.3%)	>0.999^a^
Endometrial Thickness (mm)	8.72±1.45	9.00±1.69	0.423^b^

a Chi-square test b, Independent Samples Test

## DISCUSSION

The results of our study showed that the mean endometrial thickness of patients in the TMX group was 8.72±1.45 mm and in the HRT group it was 9.00±1.69mm, and this difference was not statistically significant. These findings suggest that tamoxifen and gonadotropin can be effective for endometrial preparation in frozen embryo transfers. Our study showed no significant difference in clinical pregnancy rate, biochemical pregnancy rate, ongoing pregnancy and implantation rate between the two groups.

Tamoxifen is a selective estrogen receptor modulator (SERM) used to treat breast cancer (Pourmatroud *et al*., 2013). Previous published studies showed that use of tamoxifen is associated with endometrial proliferation in women with breast cancer. In addition, various studies have evaluated TMX in induction ovulation compared to clomiphene and gonadotropin in assisted reproduction technique (Sharma *et al*., 2018; Peyvandi & Moslemizadeh, 2006; Steiner *et al*., 200*5*; Taherian & Sadeghi, 2004). In Sherman’s study, tamoxifen (TMX) improved endometrial thickness (ET) and live birth rates in patients with thin endometria when compared to clomiphene (Sharma *et al*., 2018). Also, Peyvandi & Moslemizadeh (2006) revealed that tamoxifen is effective in inducing ovulation and pregnancy by increasing endometrial thickness and number of mature follicles due to estrogen agonist effects, and it can be considered before HMG treatment.

To date, there have been a few studies on the treatment of thin endometrium in frozen fetal cycles with tamoxifen in the literature. For the first time, Chen & Chen (2013) evaluated administration of 20 mg of tamoxifen in 3 women who showed recurrent unresponsive thin endometria with usual protocol in previous cycles, their result showed an increase in endometrial thickness up to at least 7.7 mm and all of them conceived. After a while, in a retrospective study by the same group, 61 patients with thin endometrium in the frozen embryo cycle were studied and reported that tamoxifen alone improved endometrial thickness from 6/5 to 8/8 mm (Tian *et al*., 2015).

Ke *et al*. (2018) studied the effects of tamoxifen on 226 women with a history of thin endometria in prior cycles. Common protocols were used to prepare the endometrium (ovulation induction (OI), hormone replacement treatment (HRT) and natural cycle (NC)). The patients were divided into 3 different groups of thin endometrial etiology, including patients with PCOS, history of intrauterine adhesion and uterine curettage. In this study, 20mg of tamoxifen was given in combination with estradiol. Tamoxifen cycles exhibited a significantly increased endometrial thickness in all groups (Ke *et al*., 2018). Patients with polycystic ovary have much better pregnancy outcomes in comparison to other groups. In contrast to our study, they didn’t have a control group. Also, a lower dose of tamoxifen was used compared to our study (20 mg). Our study was a clinical trial in which tamoxifen was compared with a control group given hormone therapy. The dose of our study drug was selected based on previous studies that used 40 mg TMX in combination with HMG.

Ji *et al*. (2020) conducted a retrospective study that compared pregnancy outcome after frozen-thawed embryo transfer in tamoxifen-stimulated cycles with hormone replacement treatment FET in patients with thin endometria. They found the use of TMX for endometrial preparation increased endometrial thickness that this results is in line with the finding of the previous study mentioned by Ke *et al*. (2018); and in contrast to our finding, they reported that clinical pregnancy, ongoing pregnancy and live birth in the TMX group was significantly higher than in the hormone group. In their study, the cycle would be cancelled, If there was no dominant follicle after 15 days of ovarian stimulation or endometrial thickness did not reach 7 mm (Ji *et al*., 2020). Different from the previous study, in our study, we would continue the treatment if the dominant follicle was not present and the transfer would be performed if the endometrium thickness were adequate.

The inconsistency between our findings and these studies might have resulted from variations in design, methods and our sample population. Similar to other studies, we have not seen any serious side effects in the participants. In our study, some cycles were canceled due to the non-triple line pattern of endometrial or fluid buildup within the endometrial cavity, which had not been reported in previous studies. The possible explanation for these results may be due to the difference in drug dose between the studies. We used a higher dose of the TMX in comparison to similar studies. However, further research should be undertaken to investigate the appropriate dose of TMX in FET cycles.

To the best of our knowledge, the present study appears to be the first randomized clinical trial study to compare the endometrial thickness and pregnancy outcome between TMX and hormone replacement treatment in FET. However, our study has some limitations; first, we didn’t examine any subset of patients who did not have a mature follicle separately. This may have affected our results. In addition, we couldn’t blind the patients and this might put the result at a risk of bias.

## CONCLUSION

The use of tamoxifen and gonadotropin to prepare the endometrium for FET might be as effective as the GnRH agonist group and estradiol valerate. Since tamoxifen is a cheap and available drug, it could be an appropriate alternative drug in FET cycles.
